# An Atypical Case of Poland Syndrome with Bilateral Features and Dextroposition of the Heart: In the work-up of Poland syndrome, different imaging modalities are necessary to depict the full extent of the anomalies

**DOI:** 10.5334/jbsr.1860

**Published:** 2019-07-11

**Authors:** Barbara Geeroms, Luc Breysem, Michaël Aertsen

**Affiliations:** 1 UZ Leuven, BE

**Keywords:** Case report, Poland syndrome, dextrocardia, chest wall, chest deformity

## Abstract

Poland syndrome is defined by the unilateral aplasia or hypoplasia of the sternocostal head of the major pectoral muscle and is associated with variable ipsilateral thoracic and upper limb anomalies. Most frequently, the abnormalities are unilateral and on the right side. We present an atypical case of Poland syndrome in a baby girl with bilateral chest abnormalities, mainly on the left side, and secondary dextroposition of the heart. As required in almost all cases of Poland syndrome, different imaging modalities were used to evaluate the extent of our patient’s anomalies.

## Introduction

Poland syndrome was first described in 1841 by Alfred Poland [1]. The pathophysiology is not yet fully understood. The diagnosis is often made clinically, but a thorough evaluation using different imaging modalities is required to fully depict the extent of the anomalies.

## Case Report

**Figure 1:**
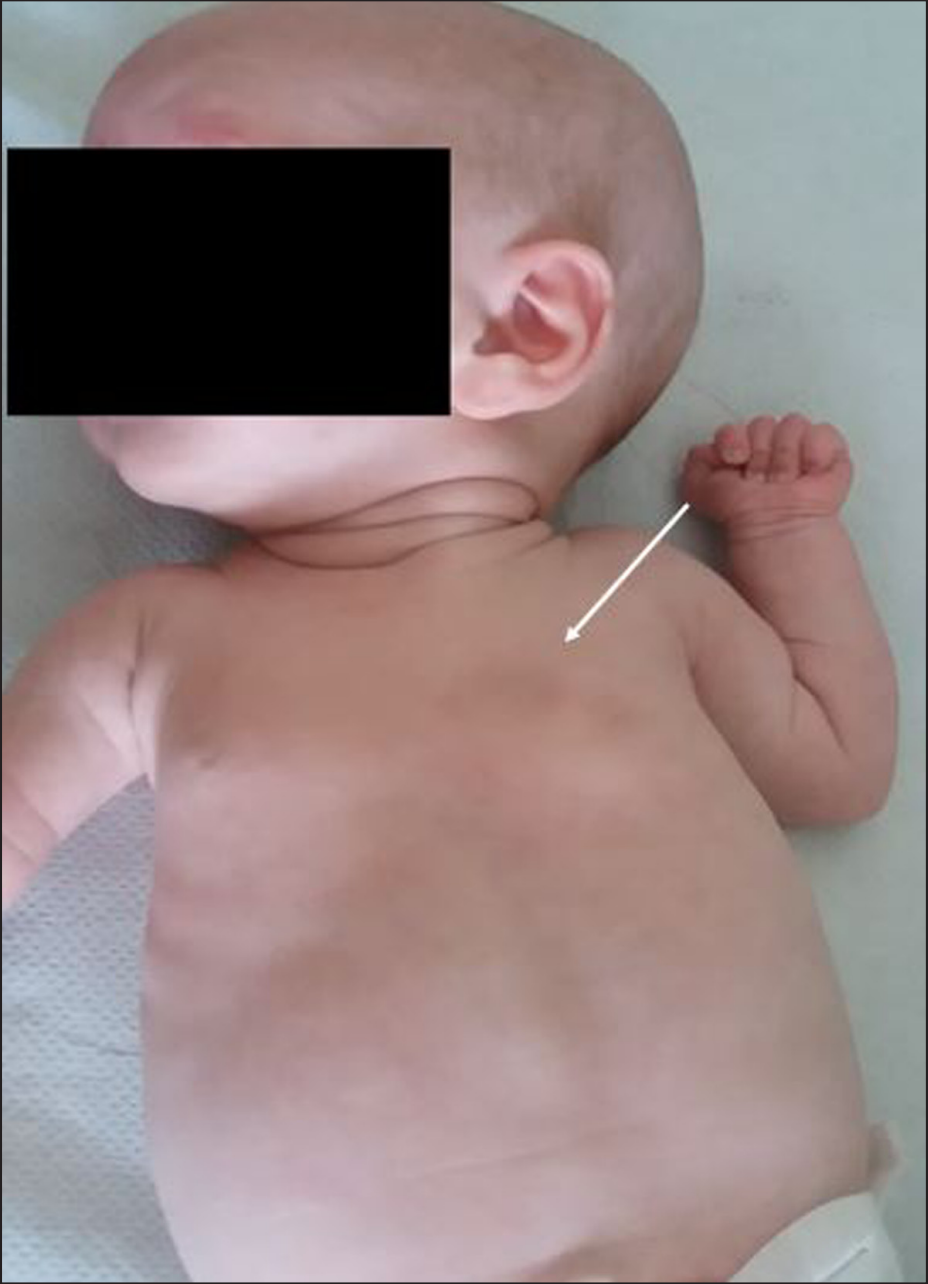
Photograph of the baby girl at 2 months of age demonstrating the asymmetry in the chest wall with impression of missing muscle and ribs 4 and 5 on the left side (*white arrow*). The bilateral gynaecomastia is also visible.

A girl was born at gestational age of 36 weeks as a member of dizygotic twins. On the first postnatal clinical examination, a focal, parasternal bulging of the left lung was noted, accentuated during crying, with impression of missing ribs 4 and 5 on the left side and missing muscle at those levels (Figure 1). She had bilateral gynaecomastia, normal extremities and no dysmorphic features. Her neurological examination was normal.

**Figure 2:**
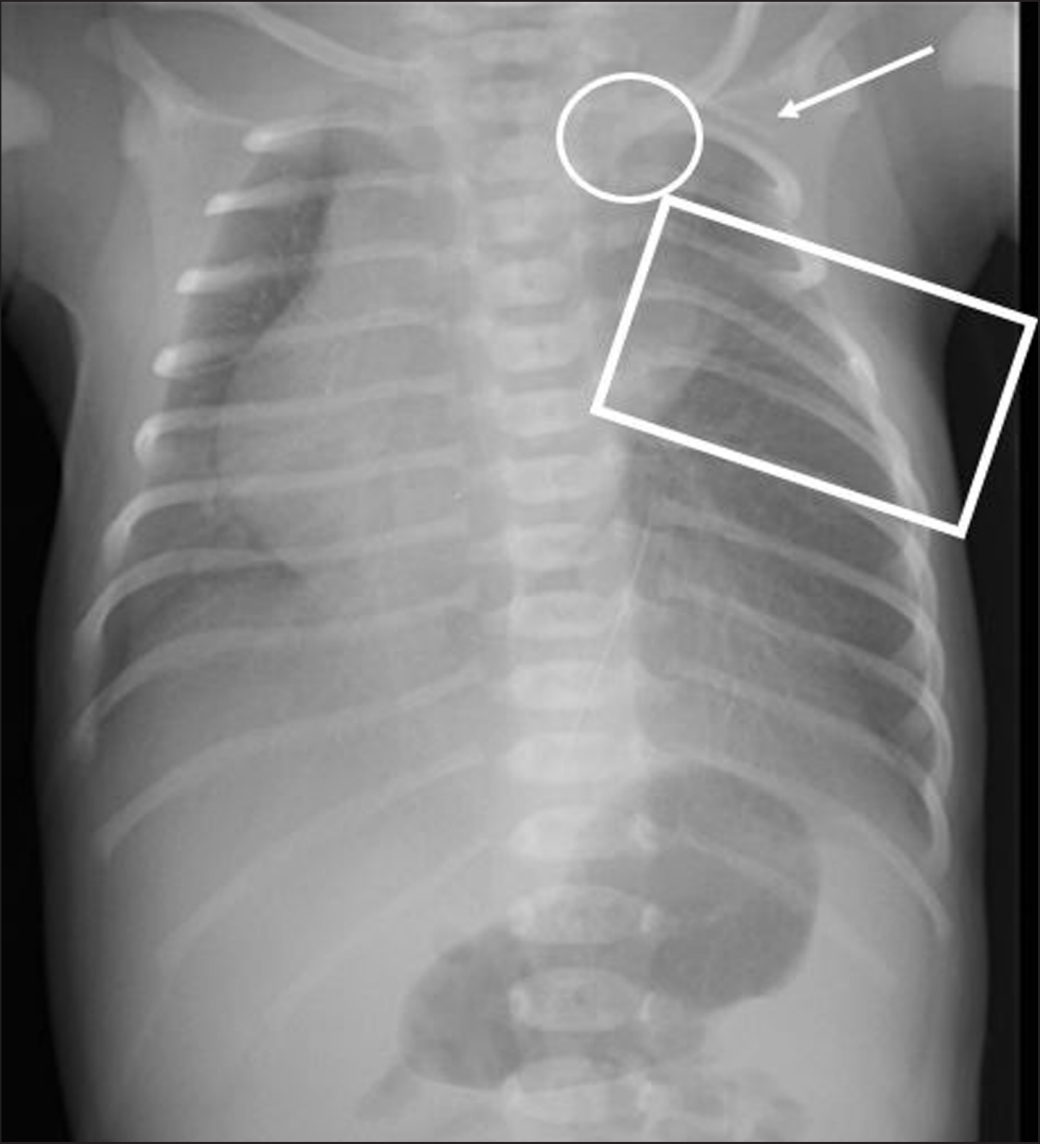
Chest X-ray at day 0 shows 11 ribs on the right side and 11 ribs plus an apparent cervical rib on the left side (*white arrow*). The intercostal spaces between ribs 4 and 6 on the left side appear narrowed (*rectangle*). The medial end of the left clavicle (*circle*) has an abnormal low position compared to the right clavicle and the cardiac silhouette is positioned on the right side.

A chest X-ray (Figure 2) showed 11 ribs on the right side and 11 ribs plus an apparent cervical rib on the left side. The medial end of the left clavicle had an abnormal low position compared to the right clavicle, and the cardiac silhouette was positioned on the right side. Based on the clinical findings and chest X-ray, the diagnosis of Poland syndrome was made.

**Figure 3:**
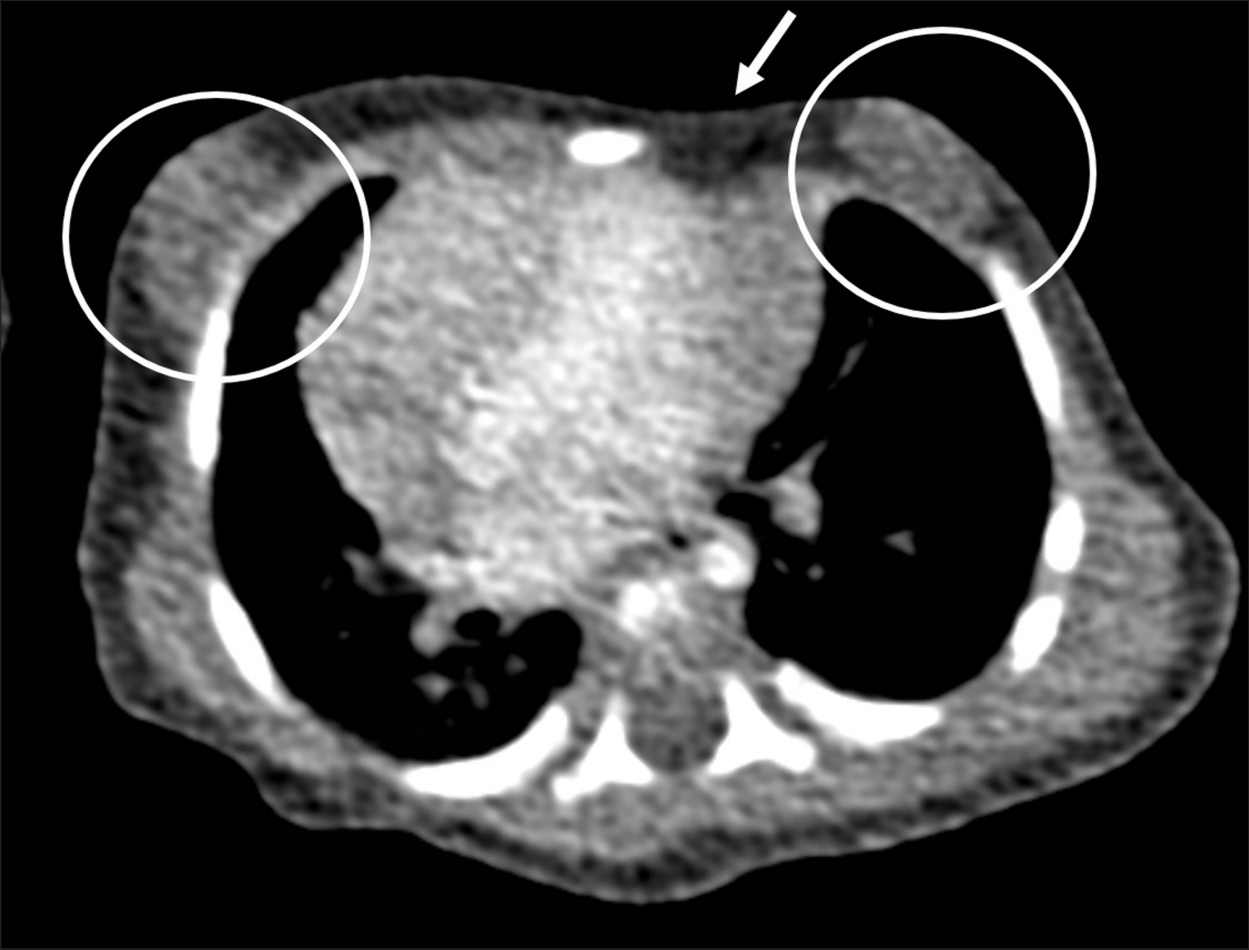
Axial CT-scan image in mediastinal window shows absence of the sternocostal head of the left major pectoral muscle (*white arrow*) with subsequent depression in the left chest wall. There is bilateral gynaecomastia (*circles*). The heart is positioned more to the right side than normal.

**Figure 4:**
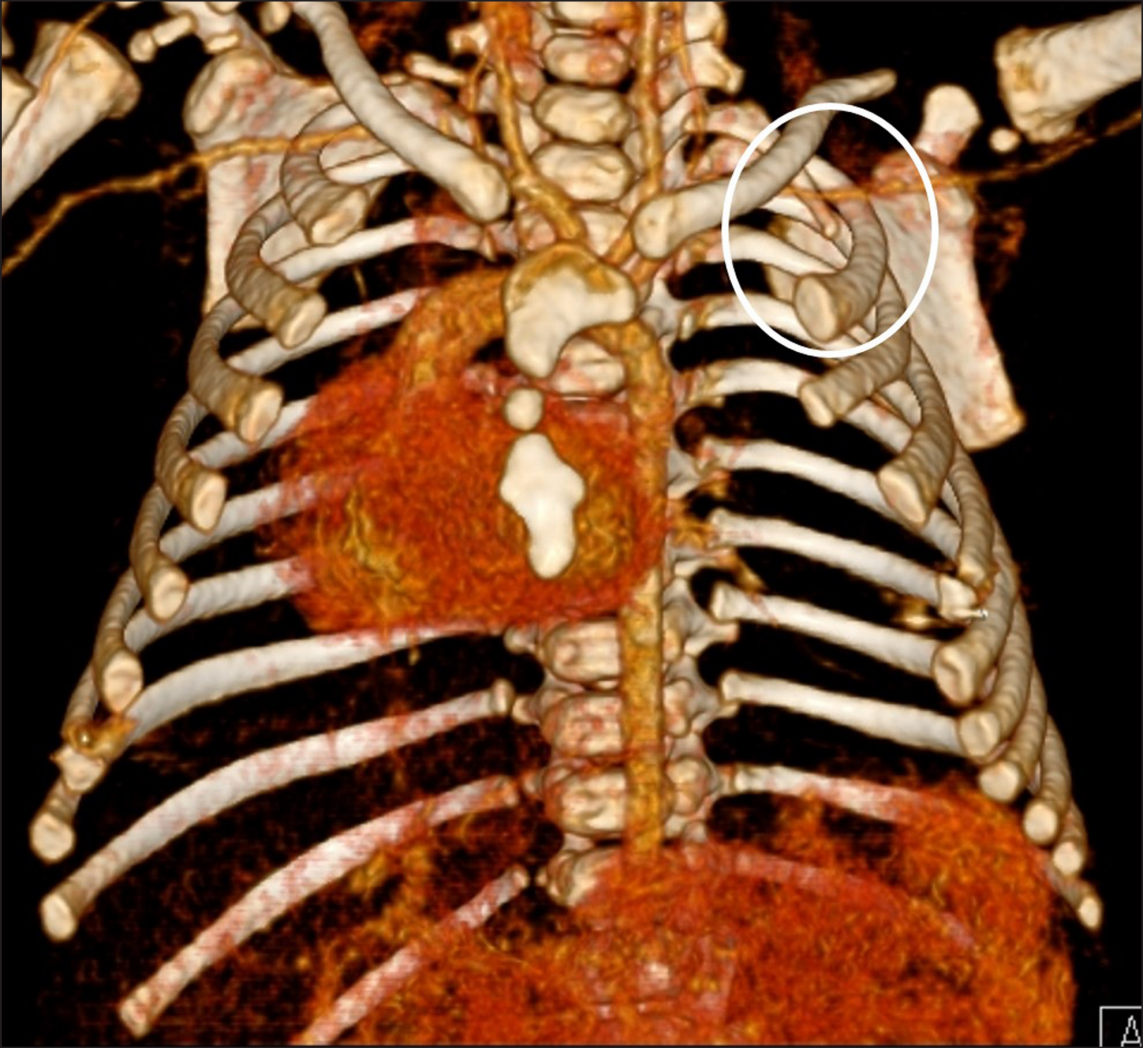
3D reconstruction of the chest CT-scan shows a dysplastic left sternal half and a dextroposition of the heart. There is a hypoplastic first rib on the left side (*circle*).

**Figure 5:**
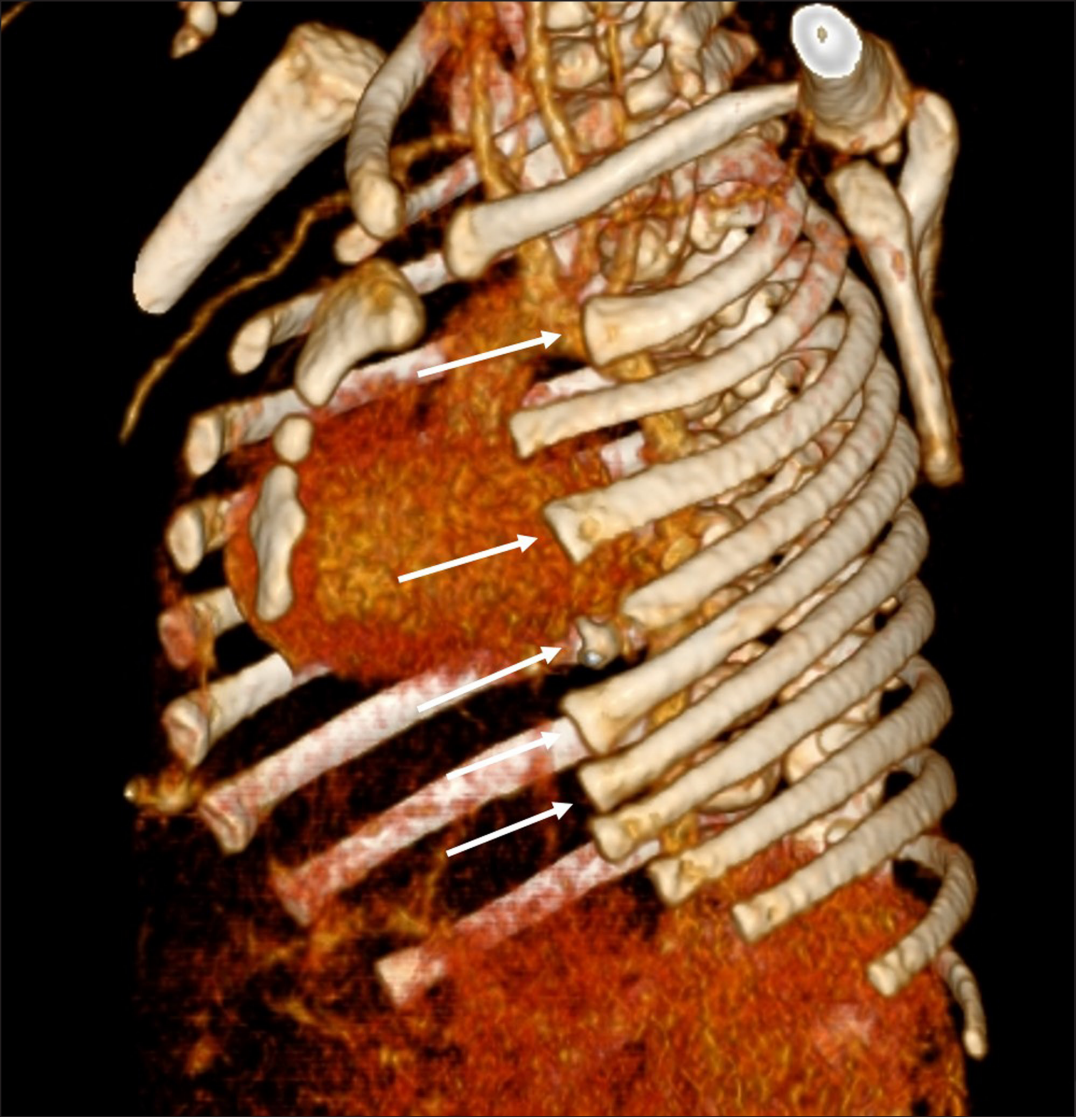
3D reconstruction of the chest CT-scan shows partial agenesis of the anterior arches of ribs 2, 4 to 7 on the left side (*white arrows*).

**Figure 6:**
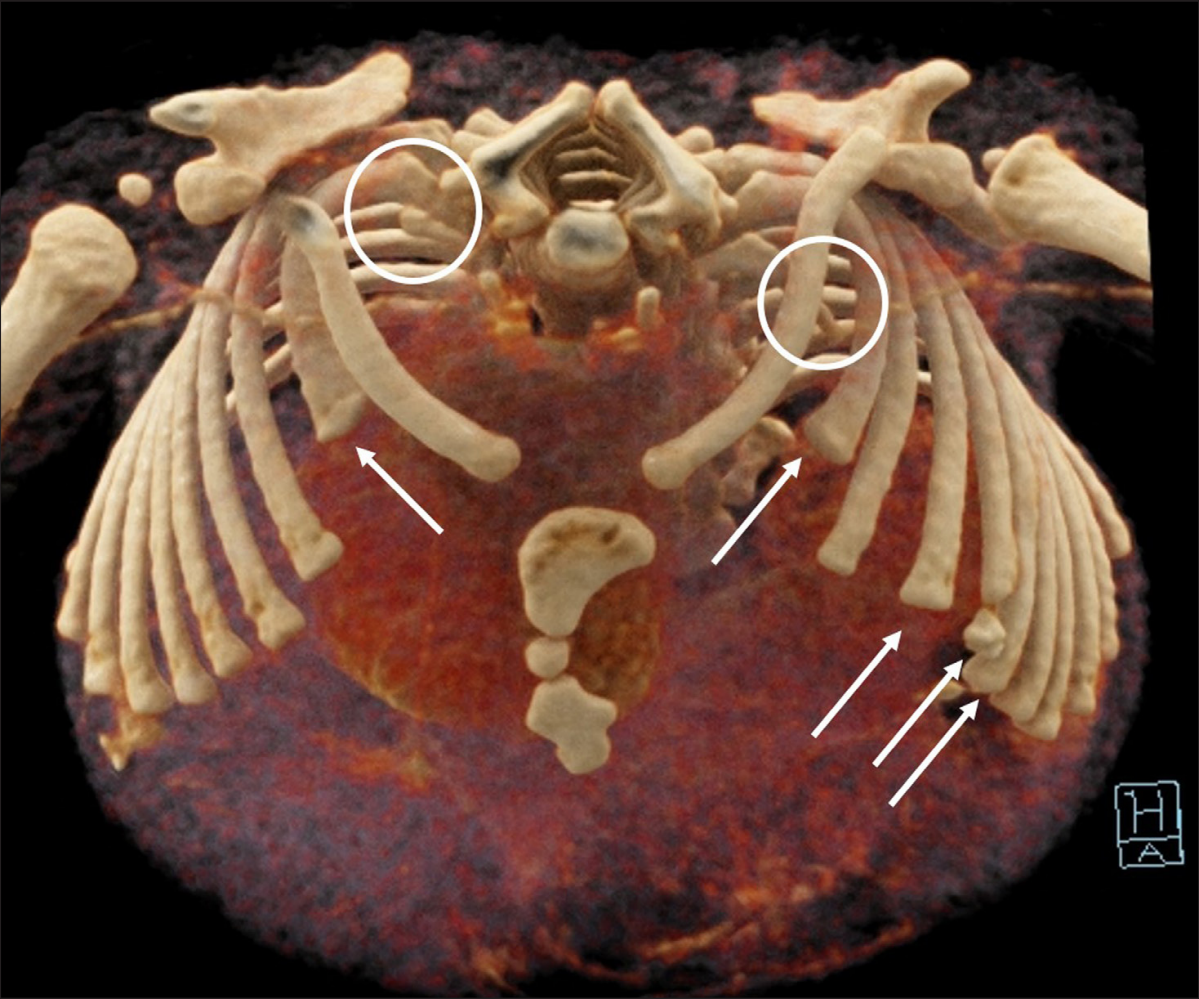
3D reconstruction of the chest CT-scan shows bilateral hypoplastic first ribs (*circles*) and a partial agenesis of the anterior arches of ribs 2 bilaterally and of ribs 4 to 7 on the left side (*white arrows*).

Abdominal ultrasound showed no associated abnormalities nor signs of situs inversus. Transthoracic cardiac ultrasound showed dextroposition of the heart with normal morphology and functionality. A chest computed tomography (CT) scan (Figures 3, 4, 5, 6) using a low-dose pediatric protocol with a CTDI of 0.33 mGy showed a dysplastic left sternal half with abnormal orientation of the left clavicle. There were 12 ribs present bilaterally, with a hypoplastic first rib bilaterally. On the left side there was partial agenesis of the anterior arches of ribs 2, 4, and 5 and, less pronounced, of ribs 6 and 7. On the right side there was partial agenesis of the anterior arch of rib 2. Secondary to these rib defects, the intercostal spaces between left ribs 4 and 8 were narrowed. The sternocostal head of the left major pectoral muscle was absent, the clavicular head was intact. Glandular breast tissue was present bilaterally. The heart was positioned in the right hemithorax with normal orientation of the apex: ‘dextroposition of the heart’. There were no vertebral defects.

The diagnosis of Poland syndrome was confirmed with bilateral, mainly left-sided chest wall abnormalities and secondary dextroposition of the heart.

## Discussion

Poland syndrome is seen in 1/20,000 to 1/30,000 of patients, and it is more frequent in males than females [1,2,3]. Most commonly (60–75% of cases) the anomalies are unilateral and affect the right hemithorax [1,2,3]. The condition is defined by the unilateral aplasia/hypoplasia of the sternocostal head of the major pectoral muscle but can be associated with other thoracic defects, including hypoplasia or aplasia of breasts and ribs, chest wall depression, and sternal anomalies [4]. Upper-limb anomalies can be associated with Poland syndrome; the most frequent is ipsilateral brachysyndactyly [1,2]. Lung herniation and dextroposition of the heart are rarely associated with it. Cardiac dextroposition has been described in 5.6 to 11.2% of patients with Poland syndrome [1]. The volume loss of the left hemithorax is only significant enough to cause displacement of the heart to the right hemithorax in patients with left-sided Poland syndrome with partial agenesis of at least two ribs [1,2]. Dextroposition of the heart differs from dextrocardia. Dextrocardia is mirroring of the heart with the heart located in the right hemithorax and the apex pointing to the right side [1] and can be associated with situs inversus. In dextroposition, the heart is positioned in the right hemithorax, but the apex is pointing to the left and there is no association with situs inversus.

The pathogenesis of Poland syndrome is not completely understood. Different hypotheses have been formulated, including teratogenic factors, like smoking and drugs [1]. The most adopted theory is the vascular hypothesis, which proposes a transient decrease in flow during the sixth week of gestation in one or more branches of the subclavian or vertebral artery [1,4]. The duration and location of the vascular disruption determines the severity and extent of the abnormalities [2,5]. This is called the ‘subclavian artery supply disruption sequence’ and also explains other anomalies that are frequently associated with Poland syndrome, such as Klippel-Feil syndrome and Sprengel deformity [1,4]. A third theory proposes genetic factors [1]. The most frequently proposed inheritance is a paradominant inheritance, in which a mutation does not translate into abnormal phenotype in heterozygous individuals, but only expresses itself when a somatic mutation occurs during embryogenesis, which leads to loss of heterozygosity. This concept explains the occasional familial occurrence of usually sporadic traits such as Klippel-Trénaunay syndrome [6]. In such cases, the mutations could be transmitted through many generations in the absence of apparent phenotype [2]. This genetic hypothesis would explain the occurrence of familial and bilateral affected cases, although these could be caused by vascular disruption of multiple arteries at different stages in development [7].

Our patient is a special case in many ways. She is female, has bilateral chest wall abnormalities, and has dextroposition of the heart secondary to the partial agenesis of multiple ribs on the left side. Her twin sister does not show any signs of Poland syndrome, although this cannot contradict a genetic hypothesis as dizygotic twins are not genetically identical.

The diagnosis of Poland syndrome is mostly made clinically. However, a thorough evaluation using different imaging modalities is required to evaluate the extent of the anomalies. So far, no clear guidelines have been published regarding the types and timing of imaging modalities that should be performed. In practice, a screening chest X-ray is often followed by a chest CT scan to depict the thoracic abnormalities. Other technical examinations are requested based on clinical findings and can include a chest MRI, X-rays of the upper limbs, echocardiography, or abdominal ultrasound. An individual approach and work-up appears to be the best way, as each patient exhibits different abnormalities.

## Conclusion

Poland syndrome with bilateral features and dextroposition of the heart is an atypical and rare presentation of a rare syndrome. A thorough clinical examination and different imaging modalities are necessary to fully depict the anomalies of patients with Poland syndrome.
